# Pharmacological Stimulation of Phagocytosis Enhances Amyloid Plaque Clearance; Evidence from a Transgenic Mouse Model of ATTR Neuropathy

**DOI:** 10.3389/fnmol.2017.00138

**Published:** 2017-05-10

**Authors:** Eleni Fella, Kleitos Sokratous, Revekka Papacharalambous, Kyriacos Kyriacou, Joy Phillips, Sam Sanderson, Elena Panayiotou, Theodoros Kyriakides

**Affiliations:** ^1^Neurology Clinic A, The Cyprus Institute of Neurology and GeneticsNicosia, Cyprus; ^2^Cyprus School of Molecular MedicineNicosia, Cyprus; ^3^Electron Microscopy and Molecular Pathology Department, The Cyprus Institute of Neurology and GeneticsNicosia, Cyprus; ^4^Bioinformatics Group, The Cyprus Institute of Neurology and GeneticsNicosia, Cyprus; ^5^Donald P. Shiley Bioscience Center, San Diego State UniversitySan Diego, CA, USA; ^6^Department of Pharmaceutical Sciences, College of Pharmacy, University of Nebraska Medical CenterOmaha, NE, USA

**Keywords:** amyloidosis, TTR, C5a receptor, macrophages, amyloid clearance

## Abstract

Hereditary ATTR V30M amyloidosis is a lethal autosomal dominant sensorimotor and autonomic neuropathy caused by deposition of aberrant transthyretin (TTR). Immunohistochemical examination of sural nerve biopsies in patients with amyloidotic neuropathy show co-aggregation of TTR with several proteins; including apolipoprotein E, serum amyloid P and components of the complement cascade. Complement activation and macrophages are increasingly recognized to play a crucial role in amyloidogenesis at the tissue bed level. In the current study we test the effect of two C5a receptor agonists and a C5a receptor antagonist (PMX53) on disease phenotype in ATTR V30M mice. Our results indicate that amyloid deposition was significantly reduced following treatment with the C5a receptor agonists, while treatment with the antagonist resulted in a significant increase of amyloid load. Administration of the C5a receptor agonists triggered increased recruitment of phagocytic cells resulting in clearance of amyloid deposits.

## Introduction

Hereditary ATTR V30M amyloidosis is a life-threatening progressive sensory-motor and autonomic peripheral neuropathy. ATTR V30M amyloidosis is caused by extracellular accumulation of misfolded transthyretin (TTR), subsequently creating insoluble aggregates of amyloid fibrils (Saraiva et al., 1984). Even though there are more than a 100 mutations causing ATTR amyloidoses, the first discovered and by far the most common is ATTR V30M, which is due to a substitution of a valine with methionine in position 30 (Val30Met) of the TTR protein (Ando et al., 2005).

There is considerable variability in the age of onset and penetrance of hereditary ATTR V30M in different countries. Epigenetic and genetic factors are believed to contribute to this variability. Complement cascade components have been shown to co-precipitate with amyloid in various forms of amyloidotic neuropathy (Hafer-Macko et al., 2000). Our group has previously identified polymorphisms in C1q to correlate with age of onset in a Cypriot cohort of ATTR V30M patients suggesting that complement C1q may be a modifier gene (Dardiotis et al., 2009). We have recently demonstrated that C1q deficient ATTR V30M mice exhibit a 60% increase in amyloid deposition compared to their C1q efficient counterparts (Panayiotou et al., 2017). C1q has also been shown to modulate beta-amyloid induced complement activation and neuronal loss in Alzheimer’s disease (Fonseca et al., 2011) as well as modulating phagocytosis of soluble pre-amyloid aggregates (Pisalyaput and Tenner, 2008). A dual role for complement has been proposed, a protective effect from early components of complement (C1q opsonizes foreign material and phagocytes) and a detrimental effect from late components such as C3a and C5a exarcebating neuroinflammation (Fonseca et al., 2011). Late component C5a is produced following the activation of any of the three pathways of the complement cascade system. The C5 complement system factor is cleaved by C5 convertases producing the C5a and C5b molecules. C5a acts as an anaphylactic molecule by attracting C5a receptor bearing cells including macrophages and neutrophils leading to a pro-inflammatory response (Mathern and Heeger, 2015). Activation of the C5a receptor in the CNS could have a detrimental role leading to neurotoxicity or a neuroprotective role through phagocytosis (Fonseca et al., 2009). However, the effects of C5a receptor activation by the C5a anaphylatoxin in ATTRV30M amyloidosis have not been elucidated yet.

More recently it has been shown that anti-serum amyloid P antibodies admnistered to AA amyloidotic mice resulted in an extensive “complement-dependent, macrophage-derived” response which reduced the amount of amyloid deposition (Bodin et al., 2010). This observation has been extended successfully in a Phase 1 study in humans (Richards et al., 2015).

The objective of the current study was to elucidate further the role of the complement in the pathogenesis of ATTR V30M amyloidosis and investigate the possibilty of therapeutically manipulating complement to eliminate amyloid deposit. We have administered two C5a receptor agonists, a full C5a receptor agonist and EP67 (a response-selective C5a receptor agonist deprived of C5a-like anaphylatoxin activity), and a C5a receptor antagonist (PMX53) and examined disease phenotype in ATTR V30M mice after 1 week. We show that treatment with the C5a receptor agonists significantly ameliorated amyloid deposition while C5a receptor antagonist PMX53 exacerbated amyloid deposits. We have also carried out mass spectrometry-based proteomic analysis, comparing the proteome of animals with the highest amount of amyloid and the lowest amount of amyloid. This analysis has shown substantial phagocytic cell activation, as well as the increased expression of proteolytic peptidases accompanying the reduction in amyloid deposition.

## Materials and Methods

### Animals and Tissue Handling

The previously published mouse model of ATTR V30M neuropathy (Kohno et al., 1997) was kindly donated by Dr. M. Saraiva. These animals are knockouts for murine TTR and have been bred to carry the human V30M mutated cDNA in the form of a transgene in a homozygous state (mTTR^−/−^hTTR^Met30+/+^). Real time PCR was used to ensure that all animals included in the experiments had the same copy number of human TTR transgenes. All animals used for the experiments were 13–14 months old since at this age amyloid deposits are well established.

All animals were kept in a regular 12-h light-12 h dark cycle and were given free access to water and food, under SPF conditions. Animals were separated in cages depending on the molecule they were treated with. Four groups of animals were included for study; one group was treated with PMX53 (kindly provided by Cephalon USA), one group with the full C5a receptor agonist molecule (purchased from Anaspec), one group with the modified C5a receptor agonist, EP67 (Sanderson et al., 2012) and finally there was a control group. Each group was comprised of six animals while the treatment period lasted for 1 week, during which each molecule was added to the animals’ water source at 20 μg/ml. The control group animals only received water without the addition of any other compound. All animal involving experiments were carried out in accordance to the 86/609/EEC Directive. Also, a project license was obtained from the Cyprus Veterinary Services approving the project and methodology (License Number: CY/EXP/P.L6/2010).

Mice were anesthetized and then euthanized using Tribromoethanol (Avertin) through IP injection at a dose of 250 mg/Kg. The animals were then exsanguinated via PBS perfusion to reduce the contribution of plasma in tissue measurements. Tissues were processed for immunohistochemistry by carrying out overnight 4% PFA fixation followed by wax embedding or were frozen and kept at −80°C for immunoblotting. Amyloid deposition assessment was confined to stomach tissue since this tissue is heavily involved in amyloid deposition at an early age in this particular mouse model of ATTR V30M neuropathy.

### Genotyping

Animals were genotyped using the PCR method. Primers for the mouse TTR gene (mTTR F 5′–CTG ACC CAT TTC ACT GAC ATT T–3′ & mTTR R 5′–CAA ATG GGA ACC TGG AAC C–3′); the human mutated transgene (hMET30 F 5′–TGCTGATGACACCTGGGAGC–3′ and hMET30 R 5′TCAGGTTCCTGGTCACTTCC–3′) were utilized for screening with annealing temperature at 58°C.

### Amyloid Plaque Visualization and Quantification

**Figure 1:**
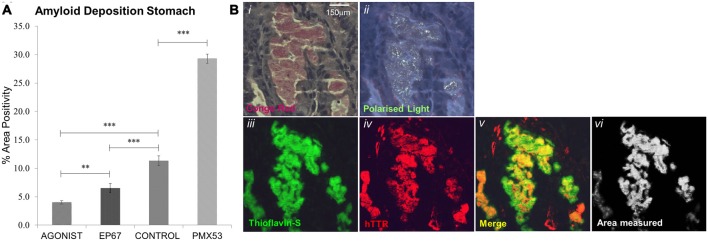
**Amyloid deposition: amyloid plaques were quantified through Thioflavin-S staining (A).** All groups exhibited significant difference from one another, with the PMX53 treated mice having the greatest amount of deposition and the full agonist treated group having the lowest recorded amount. *n* = 6/group, data presented as mean ± 1 SD. *p < 0.05, **p < 0.01, ***p < 0.001. **(B)** Amyloid plaque in the stomach that stains with Congo red and exhibits apple green birefringence **(Bi,ii)**. The same plaque stains Thioflavin-S positive **(Biii)** and is composed of human transthyretin (TTR; **Biv**). The area of co-localization of Thioflavin-S and TTR labeling appears yellow **(Bv)** and morphometric measurements are carried out with the ImageJ software **(Bvi)**. Scale bar = 150 μm.

Thioflavin S staining combined with TTR immunofluorescence were used to identify TTR specific amyloid deposits in paraffin sections obtained from stomach tissue. Paraffin sections were deparaffinized and hydrated to distilled water. Sections were then stained with Mayer’s hemeatoxylin for 5 min, washed further with distilled water and then stained with aqueous 1% Thioflavin S solution (T1892-25G) for a further 5 min and finally differentiated in 50% ethanol before been rinsed with distilled water and then mounted using the DAKO Fluorescence Mounting Medium (S3023). Thioflavin S positive deposits were further confirmed to be amyloid by Congo Red (Figures 1Bi,ii). Plaques positive for both Thioflavin S and hTTR were measured using the ImageJ software set to measure yellow (570–585 nm; Figures 1Biii–Bvi). TTR amyloid plaques were measured over the entire area of stomach section, a percentage of the surface area occupied by plaques was calculated and an average percentage obtained over five serial sections.

### Serum Elisa Method

Serum human TTR was measured using enzyme-linked immunosorbent assay (ELISA) from four animals from each of the four groups. Blood samples were collected, without sacrificing any of the animals, from the orbital sinus in the absence of anticoagulant. The samples were allowed to stand at room temperature for approximately 30 min to coagulate. They were then centrifuged at 3500 rpm for 10 min and the top layer was collected in order to obtain the serum. Samples were diluted 1/50,000 using the supplied mix diluent from the kit used (Abnova TTR Human ELISA Kit KA0495) and the procedure was carried out as outlined by the supplier. Absorbance was measured at 450 nm using a microplate reader.

### Western Blots and Densitometry

Stomach homogenate (tissue lysed with RIPA buffer and protease inhibitors under sonication) was separated via reducing SDS-PAGE and transferred onto PVDF membranes. The membranes were blocked with 5% BSA for 1 h at room temperature. The membranes were then incubated overnight at 4°C with the appropriate primary antibody. Specific antibodies were then visualized using the Super Signal West Femto Maximum Sensitivity Substrate (Thermo Fisher 34095) after incubating with the required HRP conjugated secondary antibody for 1 h at room temperature. Blots were repeated in triplicates and were visualized using the UVP bio-imaging system.

The antibodies used for immunoblotting were against: BiP (anti-rabbit Santa Cruz sc-13968 1/350), C1q (anti-rabbit Santa Cruz sc-27661 1/100), Caspase-3 (anti-rabbit Enzo Life Sciences ALX-210-806-C100 1/1000), CD68 (anti-rabbit Santa Cruz sc-9139 1/150), CD88 (anti-mouse Santa Cruz sc-53795 1/100), ELANE (anti-rabbit Abcam ab68672 1/1000), F4/80 (anti-rabbit Santa Cruz sc-25830 1/100), IL-36γ (anti-goat Santa Cruz sc-168163 1/100), Ly6G (anti-mouse Antibodies online ABIN361224 1/1000) and Properdin (anti-rabbit Santa Cruz sc-68367 1/100). The appropriate HRP conjugated secondary antibodies were used, anti-mouse (Santa Cruz SC-2031 1/5000), anti-rabbit (Santa Cruz SC-2004 1/5000).

ImageJ was used to carry out densitometry calculations, while all bands were normalized against a GAPDH loading control (Santa Cruz sc-25778 1/1000), while the same reference sample was in all westerns to allow cross-gel comparison).

### Immunohistochemistry

Paraffin sections from animals’ stomachs were deparaffinized and hydrated to distilled water. Sections were then blocked with 5% BSA solution in PBS for 1 h at room temperature and then incubated with the appropriate primary antibody overnight at 4°C. The slides were then washed and incubated with the appropriate secondary antibody for 1 h at room temperature. Finally, DAPI staining was used to label the cells’ nuclei (Sigma Aldrich D9542) before been mounted using the DAKO Fluorescence Mounting Medium (S3023). Pictures were taken using a Zeiss AXIOIMAGER M2 fluorescence microscope.

The primary antibodies used were against: BiP (anti-rabbit Santa Cruz sc-13968 1/100), Caspase-3 (anti-rabbit Santa Cruz sc-7148 1/500), CD68 (anti-goat Santa Cruz sc-7084 1/50), ELANE (anti-rabbit Abcam ab68672 1/100), Lamp-1 (anti-rabbit Cell signaling 8653 1/800) and human TTR (anti rabbit DAKO A000202 1/500). The appropriate Invitrogen Alexa Fluor 555 and 488 fluorescence secondary antibodies were used, anti-rabbit (A-21428 and A-11008 1/2000) and anti-goat (A-21432 and A-11055 1/2000).

### Mass Spectrometry-Based Proteomics

Frozen stomach tissue samples from three animals from two groups of animals (full agonist treated and PMX53 treated) were incubated in lysis buffer (10 mM Tris-HCl pH 7.4, 150 mM NaCl, 1 mM EDTA, 1% (v/v) SDS, 1X protease inhibitors) for 30 min on ice, followed by sonication for 30 s (50% pulse) using Model 150VT (Biologics Inc., Virginia, USA). Lysates were clarified by centrifugation at 12,000 rpm for 20 min at 4°C. The supernatant was collected and proteins were precipitated in tenfold excess volume of ice-cold acetone overnight at −20°C and subsequently resuspended in urea buffer (8 M urea, 50 mM ammonium bicarbonate). Protein concentration was determined using BCA protein assay. For each sample, 100 μg of protein was transferred to a new tube, reduced with DTT (10 mM final concentration) for 30 min at 60°C and alkylated with iodoacetamide (15 mM final concentration) for 15 min in dark at room temperature followed by fourfold dilution in 50 mM ammonium bicarbonate. Proteins were digested with 2 μg of proteomics grade trypsin (Roche Diagnostics GmbH, Mannheim, Germany) at 37°C for 18 h. Digestion was quenched by addition of TFA to a final concentration of 0.5%. Peptides were desalted and purified using reverse phase solid phase extraction cartridges (Sep-Pak C18, Waters, Vienna, Austria) and eluates were lyophilized using a centrifugal vacuum concentrator. Peptide pellets were re-dissolved in 1% acetonitrile, 0.1% formic acid (mobile phase A) to yield an approximate concentration of 200 ng/μL (determined by NanoDrop measurement at 280 nm). The peptide separation was performed on a Waters nanoAcquity UPLC system (Waters Co., Wilmslow, UK). Peptides were loaded onto a C18 column (Acquity UPLC M-Class, Peptide CSH, 75 μm × 250 mm, 1.7 μm, 130 Å) and eluted with a linear gradient from 5% mobile phase B (0.1% formic acid in acetonitrile) to 40% mobile phase B over 175-min. Peptides were analyzed on a Waters Synapt G2Si HDMS instrument (Waters Co., Wilmslow, UK) operated in ion mobility mode using the UDMS^E^ approach (Distler et al., 2014). Each sample analyzed in triplicate. Raw mass spectrometry data were analyzed using Progenesis QI for proteomics software (version 3.0) and were subjected to protein identification against the SwissProt mouse reference proteome database (version July 2016, 16761 sequences plus human TTR, P02766) using the MS^e^ peptide identification method. The searching parameters used were: trypsin digestion, 1 missed cleavage, FDR <4%. The identifications were refined using the following parameters: score ≥5, hits ≥2, sequence length ≥6, description not containing probable, predictive, potential or putative.

### Statistical Analyses

Statistical analysis was performed using GraphPad Prism version 5.00 for Windows (GraphPad software, San Diego, CA, USA) where one-way analysis of variance (ANOVA) followed by Tukey’s *post hoc* test was carried out. Using this information, graphical charts representing the data were prepared.

## Results

### Amyloid Deposition in the Stomach

Following administration of all three agents for 1 week, all animals were sacrificed (including untreated, age-matched control hTTRV30M animals) and amyloid deposition was examined by combined Thioflavin S staining and TTR immunofluorescence (Figure 1A). There was a 160% increase in amyloid load following the administration of PMX53 for a week when compared to the control hTTRV30M mice. Administration of the C5a receptor agonist EP67 resulted in a 42% decrease in deposited amyloid. Further amyloid reduction was recorded following administration of the full receptor agonist (65%).

### hTTR Levels in Serum and Stomach

**Figure 2:**
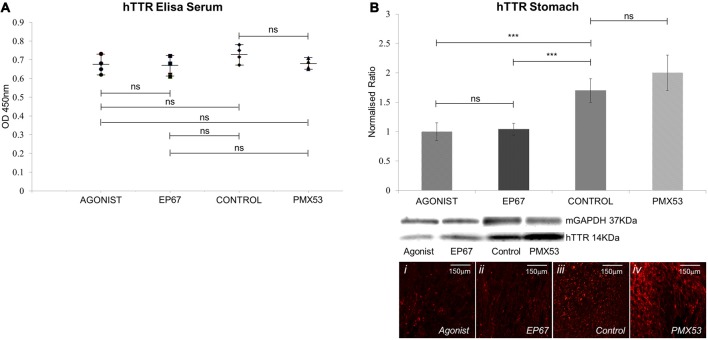
**Amount of hTTR found in the serum and stomach: (A)** TTR in the serum was quantified using enzyme-linked immunosorbent assay (ELISA). Results show that no statistical difference in the amount of circulating TTR was recorded between the four groups of mice. *n* = 4/group, data presented as mean ± 1 SD. **(B)** hTTR levels in the stomach were measured via immunoblotting. Significant decrease was observed in the mice treated with the two agonist molecules when compared to the untreated control mice. *n* = 6/group. Data presented as mean ± 1 SD. Indicative images from each group shown in **(Bi–iv)**. *p < 0.05, **p < 0.01, ***p < 0.001.

The levels of human V30M TTR were measured in the serum of all mice participating in the study using the ELISA at the end of the treatment period. Our results indicate that the amount of hTTR found in the serum remains unaffected for all four groups (Figure 2A).

The amount of pre-fibrillar hTTR was measured in stomach tissue of all animals (Figure 2B). Our results indicated significantly less hTTR between the two groups treated with the agonists and the control animals. The PMX53 group did not significantly differ from the control group.

### Phagocytic Cell Markers in Stomach Tissue

The PMX53 molecule is a known C5a receptor (CD88) inhibitor, while the other two molecules used are agonists for the receptor. Even though C5a receptors (C5R1, CD88) are ubiquitously expressed on a variety of cells, they are most prominently expressed on the surface of neutrophils and macrophages (Monk et al., 2007), therefore, identifying markers for these two cell types were used to establish their presence in the experimental animals.

**Figure 3:**
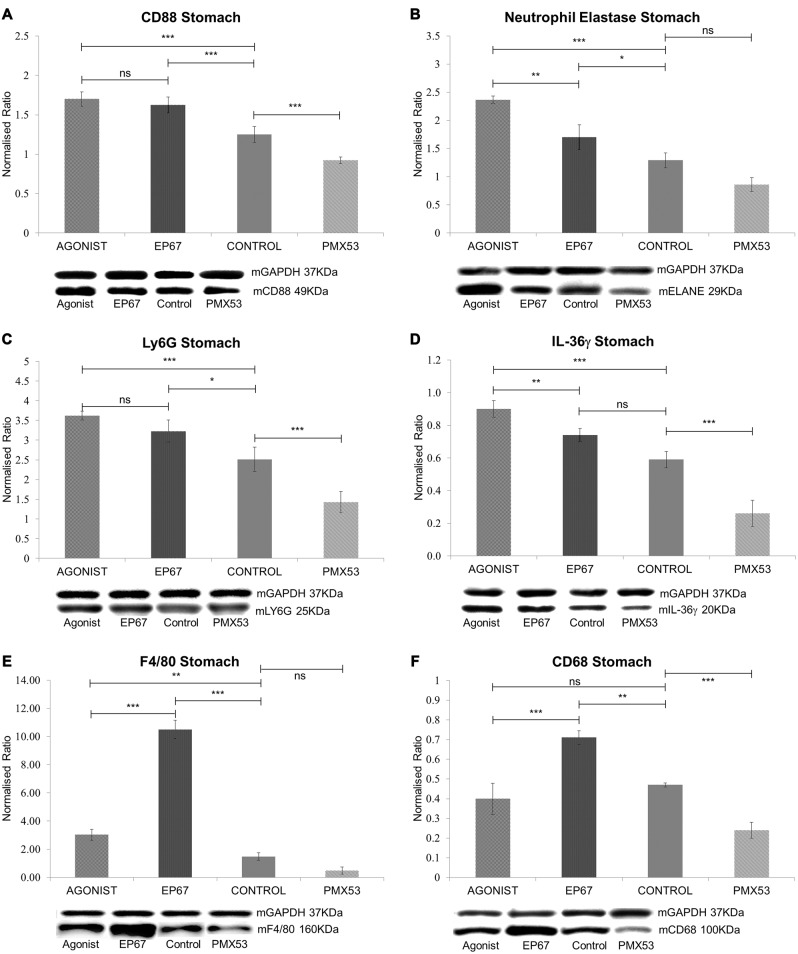
**Expression of phagocytic cell markers in stomach tissue: (A)** The expression of CD88 was measured by immunoblotting indicating a decrease in the marker in both the untreated group and the group treated with the PMX53 antagonist, while the two groups treated with the agonists exhibited the greatest level of expression. Similar effects were observed with Neutrophil elastase **(B)**, Ly6G **(C)** and IL-36γ **(D)**. Immunoblots for the macrophage specific markers F4/80 **(E)** and CD68 **(F)** however indicate their overexpression in the group of mice treated with the modified agonist molecule EP67, while the group treated with the PMX53 antagonist exhibited the lowest levels of expression for both markers. *n* = 6/group data presented as mean ± 1 SD. *p < 0.05, **p < 0.01, ***p < 0.001.

CD88, the ubiquitous C5a receptor was found to be significantly elevated in both the full agonist and EP67 groups when compared to the control animals, whereas the receptor was severely decreased in the animals treated with PMX53 (Figure 3A).

Neutrophil elastase (ELANE) and Ly6G are both well characterized markers of neutrophils (Talukdar et al., 2012; Amsalem et al., 2014). ELANE is a chymotrypsin like serine proteinase which is mainly secreted by neutrophils during inflammation in order to induce the clearance of bacteria and host tissue (Belaaouaj et al., 2000). ELANE is also very similar to other immune system cytotoxic serine proteases such as granzymes and cathepsin G (Thomas et al., 2014). Our results show that ELANE is highest in the group of mice treated with the full agonist molecule, which retains its anaphylactic properties, while it is lower in the group treated with the agonist EP67 which activates C5aR receptors on macrophages but less so on neutrophils (Figure 3B). The PMX53 treated animals were found to express the lowest levels of ELANE even though the change was not statistically significant when compared to the untreated control group.

Ly6G is s neutrophil specific marker which has been previously used to deplete neutrophils in mice (Pillay et al., 2013). Our data indicate a similar pattern as with ELANE, where the groups treated with the agonist molecules exhibit the highest level of Ly6G, while the mice treated with the PMX53 antagonist molecule exhibit the lowest amount of Ly6G detected (Figure 3C).

Interleukin 36γ (IL-36γ) has been found to be up-regulated in a number of inflammatory diseases and is believed to be expressed by both neutrophils and macrophages (Bozoyan et al., 2015; Kovach et al., 2016; Macleod et al., 2016). Our results also indicate that IL-36γ is highest in the group of mice treated with the full agonist and the lowest in the group treated with the PMX53 molecule (Figure 3D).

The murine F4/80 (EGF-like module-containing mucin-like hormone receptor-like 1, Emr1 homolog) is a well-known marker of murine macrophage populations (Austyn and Gordon, 1981), while CD68 is found to be expressed on all macrophages (Murray and Wynn, 2011). The PMX53 treated mice exhibited the lowest levels of both macrophage markers, while both groups of mice treated with the agonist molecules displayed greater expression of the two markers when compared to the untreated control (Figures 3E,F). However, the mice treated with EP67 display the greatest levels of macrophages, even when compared to the group treated with the full agonist.

Results yielded from the liquid chromatography—tandem mass spectrometry (LC-MS/MS) analysis comparison between the group with the highest amyloid deposition (PMX53 treated) and the group with the lowest amount of amyloid (full agonist treated) revealed a number of macrophage and neutrophil related markers which were highly expressed in the animals with the lowest amount of amyloid (Supplementary Table S1).

### Immunofluorescence with Neutrophil and Macrophage Markers

In order to examine the co-expression of the macrophage and neutrophil markers with the amyloid plaques serial deparaffinized stomach sections were immunostained with antibodies specific for CD68, neutrophil elastase/ELANE, hTTR and Thioflavin-S.

**Figure 4:**
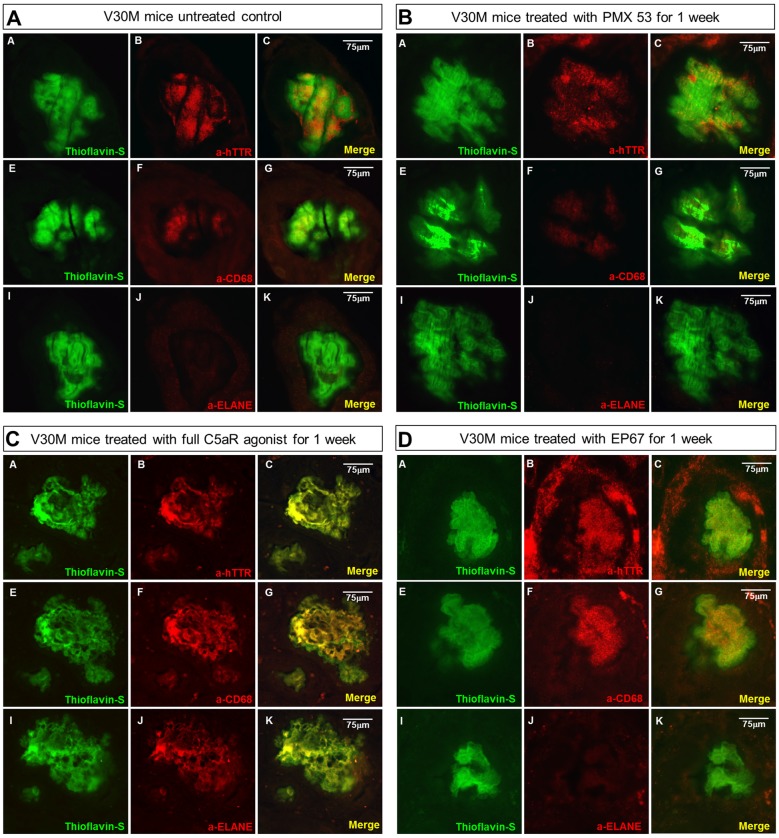
**Amyloid plaque infiltration by macrophages and neutrophils: serial sections stomach sections from mice representing the four groups of mice were stained with Thioflavin-S, α-TTR, the pan-macrophage marker α-CD68 and the neutrophil marker α-Neutrophil elastase (ELANE).** Immunofluorescence on the stomach tissue from the untreated mouse indicates the complete absence of neutrophils from the plaque, while there is some co-expression with CD68 **(A)**. A similar pattern was observed with the mouse treated with the PMX53 antagonist molecule **(B)**, while complete co-localization with both CD68 and ELANE was observed in the mouse treated with the full agonist molecule **(C)**. Immunofluorescence on the mouse treated with the modified agonist EP67 however revealed complete plaque co-localization with CD68 and the complete absence of ELANE from the region **(D)**. Scale bar = 75 μm.

The untreated group displays minimal co-localization of α-CD68 with the Thioflavin-S/hTTR positive plaques, while there is no co-localization with α-ELANE (Figure 4A).

The sections from the mice treated with the C5a antagonist PMX53 exhibit an even lower level of α-CD68 co-localization, probably indicative of the fact that there’s a decreased recruitment due to inhibition of the receptor (Figure 4B).

In the group treated with the full agonist there was complete plaque co-localization with both α-CD68 and α-ELANE (Figure 4C). The EP67 treated group however exhibited mainly co-localization with α-CD68 positivity with a much less neutrophil recruitment (Figure 4D).

### Complement Markers in Stomach Tissue

The complement system has been previously shown to be involved with amyloidogenesis and the pathogenesis of the disease. Therefore, markers for both the classical and alternative complement system were analyzed using immunoblotting.

**Figure 5:**
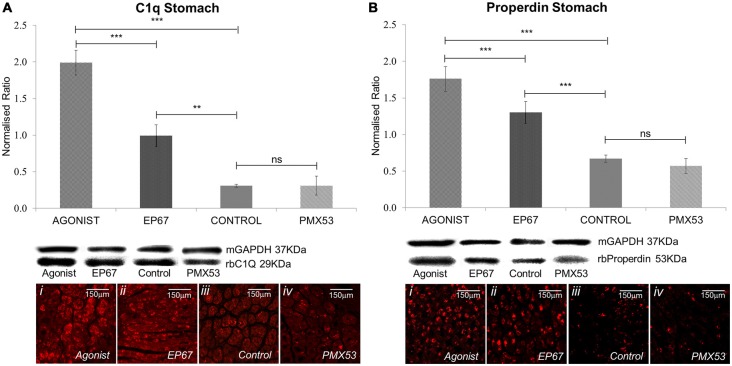
**Expression of complement markers: the expression of the classical complement pathway protein C1q was measured by immunoblotting in stomach tissues of all the animals from the four groups of mice (A).** The group treated with the full agonist molecule exhibited the highest levels of C1q which was statistically significant when compared to the rest of the groups, while the mice with the modified agonist also exhibited a significant increase when compared to the untreated control group. Similar effects were observed via immunofluorescence. Properdin, the alternative complement pathway marker, also presented an analogous pattern so that the group of mice treated with the two agonists also expressed the greatest amount of the protein **(B)**. *n* = 6/group, data presented as mean ± 1 SD. B&D Scale bar = 150 μm. *p < 0.05, **p < 0.01, ***p < 0.001.

The classical complement pathway is initiated through the C1q molecule. C1q itself is produced by peripheral tissue phagocytic cells (Petry et al., 1991). The group of mice treated with the full agonist molecule express the greatest amount of C1q; even though the mice treated with the EP67 molecule also exhibit elevated amounts of C1q when compared to the untreated control animals. On the contrary, the animals treated with the C5aR inhibitor, PMX53; do not display any significant difference from the control animals (Figure 5A).

Properdin (factor P) is a unique positive regulator of complement activation which functions by stabilizing the alternative pathway convertases (Smith et al., 1984) so that it may be used as an alternative complement pathway marker.

Our results indicate the greatest amount of properdin in the animals treated with the full agonist, followed by the EP67 treated group (Figure 5B). The PMX53 treated mice express the same amount of properdin as the control group.

These results were also corroborated through the LC-MS/MS analysis, where the complement cascade proteins, along with properdin, were found to be higher in the group of mice treated with the full agonist molecule when compared to the group of mice treated with the PMX53 molecule (Supplementary Table S1).

### Stress and Apoptosis Markers in Stomach Tissue

**Figure 6:**
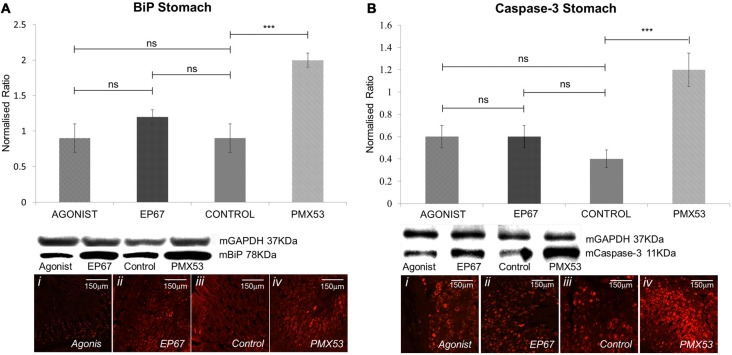
**Expression of stress markers: the expression of BiP was measured by immunoblotting (A)** in the stomach tissues of all animals from the four groups. While the animals from the groups treated with the two agonists and the untreated group displayed comparable levels of BiP, the group treated with the PMX53 antagonist exhibited significantly greater amounts of the cellular stress marker. Comparable effects were observed via immunostaining. Similarly, immunoblotting using the apoptotic marker Caspase-3 displayed a significantly increased expression in the group of mice treated with PMX53 **(B)**. *n* = 6/group, data presented as mean ± 1 SD. B&D Scale bar = 150 mm. *p < 0.05, **p < 0.01, ***p < 0.001.

The presence of extracellular amyloid deposits and pre-fibrillar hTTR species have been shown to increase endoplasmic reticulum stress through the activation of the classical unfolded protein response pathways in tissues not specialized in hTTR synthesis (Teixeira et al., 2006; Macedo et al., 2007). Our results indicate that the mice treated with PMX53 possess the highest levels of BiP which is in accordance with the high levels of amyloid deposits. The groups treated with the agonists exhibit significantly less amount of BiP than the PMX53 mice even though they do not appear to be lower than the control untreated mice (Figure 6A).

Immunostaining of nerve biopsies from hereditary V30M patients carried out with an activated caspase-3 specific antibody has shown that expression of this apoptotic marker increases as the disease progresses (Sousa et al., 2001). We have also observed a significant increase in Caspase-3 in the group with the highest amyloid load (PMX53 treated) as compared to the other groups, even though no significant difference was recorded between the agonists treated groups and the control group (Figure 6B).

The LC-MS/MS analysis has revealed a great number of ER related stress markers which appear greatly increased in the PMX53 treated group of mice, as well as a number of Caspases and apoptotic markers (Supplementary Table S2).

### Lysosomal Marker in Stomach Tissue

**Figure 7:**
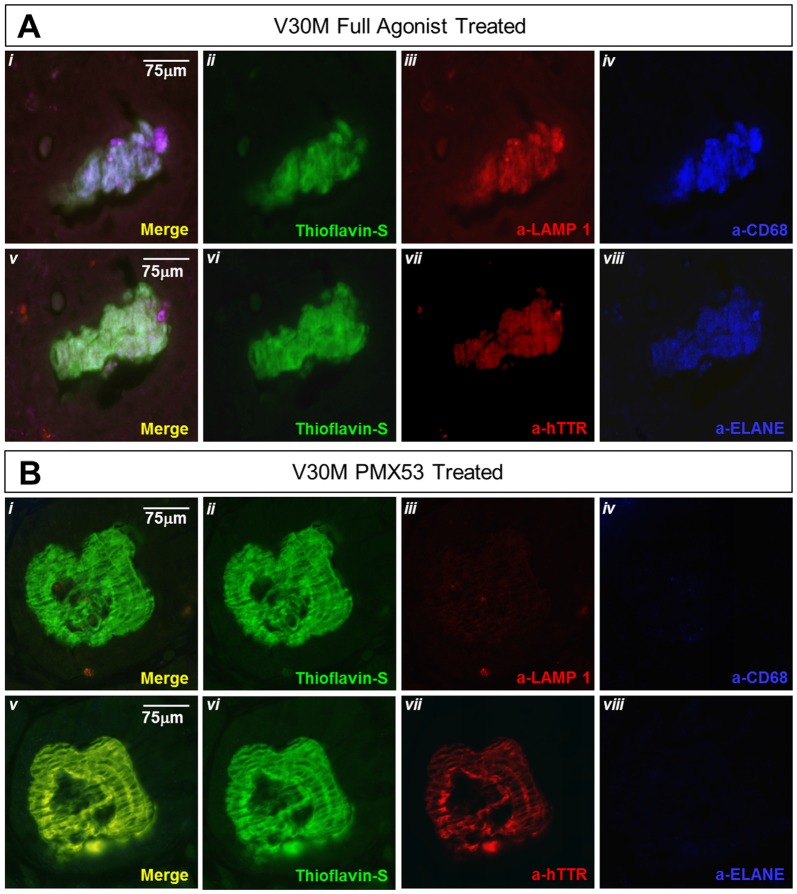
**Amyloid plaque co-localization with the lysosomal marker: immunofluorescence of serial stomach sections from the group treated with the full agonist molecule (A)** and the PMX53 antagonist **(B)** show the complete co-expression of the amyloid plaque with Lamp-1 **(Ai)** in the mouse treated with the agonist as opposed to the antagonist treated mouse which presents complete absence of the lysosomal marker in the vicinity of the plaque **(Bi)**. Sections were co-stained with Thioflavin-S, a-Lamp-1 and a-CD68 **(Ai-iv and Bi-iv)** and Thioflavin-S, a-hTTR and a-ELANE **(Av-viii and Bv-viii)**. Scale bar = 75 μm.

Lysosomal-associated membrane protein 1 (Lamp-1) is a glycoprotein known to primarily reside across lysosomal membranes (Carlsson and Fukuda, 1989), also Lamp-1 may be expressed on the cell surface following lysosomal fusion with the cell membrane during phagocytosis (Kima et al., 2000). During the formation and maturation of the phagosome, Lamp-1 will specifically become localized on the phagosomes (Sugaya et al., 2011), so that, expression of Lamp-1 signifies the final steps of activated phagocytosis. Serial deparaffinized stomach sections from a mouse treated with the full agonist and EP67 exhibit the complete co-localization of α–Lamp-1 with α–CD68 and α–ELANE. On the contrary, the PMX53 treated mouse does not exhibit any co-localization with ELANE, CD68, or LAMP-1(Figure 7A). On the contrary, sections from an animal treated with PMX53 display no plaque co-localization with Lamp-1 (Figure 7B).

### Liquid Chromatography–Tandem Mass Spectrometry Analysis

**Figure 8:**
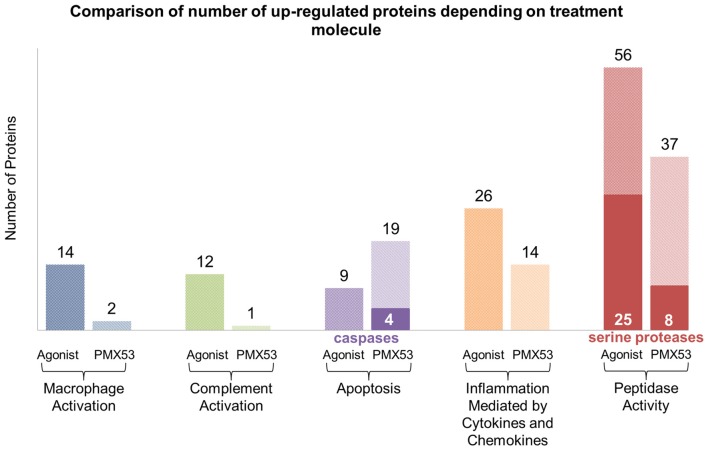
**LC-MS/MS clustering: protein functional clustering was carried out through the Panther tool between the group of mice treated with the full agonist molecule and the PMX53 antagonist.** The group of mice treated with the full agonist displayed an increase in the expression of markers related with macrophage and complement system activation. Furthermore, an upsurge in the expression of peptidases and factors involved with inflammation was observed. The group treated with the antagonist exhibited a greater expression of caspases and other proteins involved in the apoptosis pathways.

Considering that the animals treated with the full agonist molecule exhibited the least amount of deposited amyloid, and the animals treated with the PMX53 molecule had the highest amount of amyloid recorded, these two groups were compared using label-free mass spectrometry-based proteomic approach. Overall, a total of 3154 quantifiable proteins were identified. The expression of most of the markers examined using immunoblotting, as well as other related markers, was also confirmed through this technique (Supplementary Tables S1, S2). Furthermore, the data obtained through the LC-MS/MS technique was further analyzed using the Panther tool in order to identify clusters of proteins involved with specific functions in the two extreme groups of mice (Thomas et al., 2003; Mi et al., 2010). The most relevant results from this analysis have been summarized in Figure 8, while the specific proteins can be found in Supplementary Tables S3–S7. Also, the full list of obtained proteins along with their confidence scores can be found in Supplementary Data Sheet 1. A greater number of proteins involved with macrophage and complement activation were found to be highly expressed in the mice treated with the full agonist molecule. Similarly, a greater number of proteins associated with inflammation mediated by cytokines and chemokines, as well as peptidases were also found in the group treated with the full agonist molecule. However, the animals treated with PMX53 appeared to express a greater number of apoptosis related proteins when compared to the full agonist treated group.

## Discussion

Currently there is a variety of approaches, either in the clinic or in clinical trials, for treating ATTR V30M amyloidotic neuropathy; liver transplantation, TTR stabilizers (Tafamidis, Diflunisal), inhibitors of TTR translation (anti-sense oligonucleotides, silencing RNAs), drugs that interfere with amyloid dynamics at the tissue level (doxycycline/TUDCA). There is however an unmet need that arises from the fact that already formed amyloid deposits continue to form foci for further deposition of normal TTR giving rise to further organ damage such as the heart and the kidneys. In addition, amyloidogenesis in certain tissue beds such as the eye and the brain has not yet been effectively addressed partly due to issues of access of the various treatments to the relevant tissue beds. More recently, antibodies against serum amyloid P or in the case of ATTR V30M amyloidosis, a cryptic TTR epitope visible only when TTR is in its monomeric form, are beginning to be explored as possible treatments (Richards et al., 2015; Hosoi et al., 2016). Both of these antibodies appear to enhance phagocytosis by macrophages. Complement participation has also been demonstrated with the anti-serum amyloid P antibody *in vivo*. In the current study we provide evidence that amyloid phagocytosis can be modulated by pharmacological manipulation of the C5a receptor and in the absence of any antibodies perhaps offering a more generic and convenient treatment option for the amyloidoses.

Agonist and antagonists of the C5a receptor molecule are constructed based on the C terminus of the C5a molecule (Higginbottom et al., 2005). Agonists can be used to activate the C5a receptor bearing cells such as macrophages and neutrophils in order to induce the release of proinflammatory agents, thus activating an inflammatory response (Short et al., 1999). The C5a molecule is considered an anaphylatoxin based on its propensity to induce mast cell, basophil and neutrophil degranulation (Lee et al., 2008).

The full agonist molecule of the C5a receptor used here ((N-Methyl-Phe)-Lys-Pro-d-Cha-Cha-d-Arg-CO_2_H) is a peptide analog of the last six C-terminal residues of the C5a molecule (Higginbottom et al., 2005). In essence, this molecule retains its anaphylactic activity by not only activating macrophages but also neutrophils. EP67 (Thr-Ser-Phe-Lys-Asp-Met-Pro-(MeLeu)-D-Ala-Arg), a conformationally-restricted decapeptide of the last ten amino acids of the C5a molecule contains an altered structure, which is accommodated by C5a receptors expressed on antigen presenting cells such as macrophages (Sanderson et al., 2012; Hanke et al., 2013). The C5a receptor antagonist PMX53 (AcF-[OP(D Cha) WR]) is a small cyclic peptide molecule which binds the C5a receptor suspending its downstream function (Woodruff et al., 2006; Lobato and Rocha, 2012).

Our results show that while administering the PMX53 compound results in a 160% increase of fibrillar amyloid deposition (both the full agonist and EP67 result in the decrease of amyloid deposition by 65% and 42%, respectively) following 1 week of oral administration through the animals’ drinking water. Therefore, inhibition of the C5a receptor (CD88) results in an increase in amyloid deposition, whereas enhancing the action of the receptor induces a considerable decrease. This decrease is even more substantial following the administration of the full agonist molecule which recruits both macrophages and neutrophils (Baik et al., 2014).

The fact that the amount of circulating hTTR protein in the serum remains unchanged in all four groups is expected since liver production of TTR is not expected to be affected, while the changes in tissue handling of TTR as a result of the administration of the three types of molecules for only a week would be too short to influence serum levels. However, there was a significant decrease in the amount of prefibrillar hTTR detected in the stomach tissue of animals receiving the C5 receptor agonists when compared to control presumably due to increased phagocytosis of prefibrillar hTTR. The PMX53 group mice exhibited no significant rise in prefibrillar hTTR, compared to control, as might be expected due to reduced phagocytosis of prefibrillar hTTR (Misumi et al., 2013; Suenaga et al., 2016). This is most likely explained by a shift to a higher rate of amyloid formation driven by higher prefibrillar hTTR.

The marked decrease in amyloid load observed with the animals treated with the C5a receptor agonists is accompanied by a remarkable increase in neutrophil and macrophage markers. EP67 predominantly targets the macrophage populations, while the full agonist, which also targets neutrophils, produces significantly more IL-36γ perhaps indicative of phagocytic capacity (Sanderson et al., 2012; Hanke et al., 2013). The extra 23% decrease in amyloid observed in the full agonist treated mice is probably a direct effect of neutrophil activation and recruitment. Immunofluorescence labeling of the plaques shows no co-localization with neutrophils except in mice treated with the full C5a receptor agonist (Figure 4C). Interestingly, this is the group which exemplifies the greatest reduction in amyloid load, indicating a capacity of neutrophils to clear amyloid (Baik et al., 2014). Moreover, amyloid plaques in animals treated with the two C5a receptor agonists exhibit increased expression of the lysosomal marker Lamp-1, signifying that the recruitment of macrophages and neutrophils does ultimately lead to activated phagocytosis of the amyloid plaque. Additionally, the PMX53 treated group of animals exhibited the lowest expression of both neutrophil and macrophage markers as might be expected due to the inhibition of the C5a receptor (Brennan et al., 2015; Gupta and Kaplan, 2016).

The complement pathway is involved in innate immunity and C5a is one of the final effector molecules produced. C5a is imperative in tissue clearance through the recruitment of inflammatory cells. Mice treated with the C5a receptor agonists express a greater amount of C1q than both the control and the PMX53 treated mice. Neutrophils are known to possess C1q receptors which in turn enhance the expression of the CR3 receptor (Eggleton et al., 1994), an integral part of the innate immune response. Therefore, the significant increase in C1q, observed especially in the group treated with the full C5a receptor agonist, is probably due to the presence of neutrophils. Neutrophils also activate the alternative complement pathway and release C5 fragments, which further amplify the neutrophil pro-inflammatory response, acting in a positive feedback loop (Camous et al., 2011). Furthermore, macrophages have also been shown to activate the alternative complement pathway by activating C3 (Schorlemmer et al., 1977), explaining the increase in properdin in the groups treated with the C5a receptor agonists. The PMX53 treated mice however did not appear to produce less properdin than the control group of animals, probably a response to the increased amyloid deposition where several complement components co-localize (Reichwald et al., 2009). This evidence is also corroborated by the LC-MS/MS analysis, which indicates the massive up-surge of both the classical and alternative complement cascade in relation to the PMX53 treated group (Supplementary Table S1).

The LC-MS/MS data reveal that a greater number of peptidases, and more specifically serine proteases, become up-regulated in the full agonist treated group when compared to the PMX53 treated animals which exhibit the greatest amyloid load. Evidence from work carried out on amyloid β peptides in Alzheimer’s brains, identifies peptidase and protease mediated cleavage as a possible clearance mechanism in the catabolism of amyloid plaques (Matsumoto et al., 1995; Ogawa et al., 2000; Hersh, 2003; Malito et al., 2008). Neutrophils are known to release a number of serine proteases which induce chemokine and cytokine release as well as proteolytic cleavage (Kessenbrock et al., 2011; Meyer-Hoffert and Wiedow, 2011). Furthermore, neutrophil elastase (ELANE), a serine protease secreted by neutrophils has been shown to preferentially activate IL-36 yielding the three by-products IL-36α, IL-36β and IL-36γ triggering further inflammatory response. We present data, via immunoblotting and LC-MS/MS analysis demonstrating the significant increase of IL-36γ in animals treated with the C5a receptor agonists vs. the animals treated with the PMX53 inhibitor.

Both apoptosis and cellular stress have been shown to increase along with extracellular TTR amyloid deposition (Macedo et al., 2007). We observe both by immunoblotting and LC-MS/MS analysis, that the groups of animals treated with the C5a receptor agonists do not have lower levels of apoptosis or cellular stress when compared to the control despite a reduction in amyloid load. This is perhaps partly due to the presence of macrophages and neutrophils *per se* since both are involved in inflammatory pathways which do evoke the ER stress response pathway (Gotoh et al., 2011). Similarly, macrophages have the ability to release Fas ligands, thus increasing extrinsic-signal triggered apoptosis (Brown and Savill, 1999) but also the ability to induce chronic inflammation leading to further apoptosis (Diez-Roux and Lang, 1997; Gregory and Devitt, 2004).

In summary our data show that while inhibition of the C5a receptor results in the significant increase of amyloid load, activation of the C5a receptor results in a substantial reduction of amyloid deposits. Measurable effects were seen with only 1 week oral intake and with no visible side-effects on the mice. The full C5a receptor agonist molecule, retaining its full ability to activate neutrophils, had a greater impact in reducing the amyloid load. The long term safety of the of both C5a receptor agonists will need to be assessed in the mouse model whether on a continuous or intermittent basis.

The role that neutrophils and macrophages may hold in activating amyloid clearance mechanisms also needs to be addressed in the context of other amyloidoses models. It appears that migration towards the plaque may not be determined by the plaque inducing peptide *per se*, but rather on the generic amyloid fibrillar formation since experiments have shown that individual αβ monomers do not have the ability to attract peripheral phagocytic cells (Baik et al., 2014). Therefore, activating the phagocytic immune response through the complement cascade may target generic amyloidoses.

The therapeutic exploitation of these small molecules as generic treatment in the amyloidoses will no doubt be both more challenging and rewarding.

## Author Contributions

EF: data acquisition, data analysis and interpretation. KS: data acquisition and data analysis. RP: sample preparation, data acquisition. KK: study conception, manuscript critical revision. JP and SS: study design, manuscript critical revision. EP: study conception and design, data acquisition, data analysis and interpretation, drafting of manuscript. TK: study conception and design, data interpretation, drafting of manuscript.

## Funding

This study was funded by the Cyprus Institute of Neurology and Genetics under the Telethon grants (Code: 33173126).

## Conflict of Interest Statement

The authors declare that the research was conducted in the absence of any commercial or financial relationships that could be construed as a potential conflict of interest.
