# Supplementary material for: Pharmacological Stimulation of Phagocytosis Enhances Amyloid Plaque Clearance; Evidence from a Transgenic Mouse Model of ATTR Neuropathy
Source: Front Mol Neurosci. 2017 May 10;10:138. doi: 10.3389/fnmol.2017.00138 (PMC5423984; doi:10.3389/fnmol.2017.00138)
Supplement: Supplementary file 1 [file Table_1.docx]

| Accession | Confidence score | Anova (p) | Max fold change | Highest mean condition | Lowest mean condition | Description |
| --- | --- | --- | --- | --- | --- | --- |
| *Neutrophil related makers* | | | | | | |
| P05208 | 154.5 | 2.6E-05 | 34.80 | Agonist | PMX53 | Chymotrypsin-like elastase family member 2A -Cela2a- |
| Q91X79 | 146.6 | 6.7E-05 | 4.86 | Agonist | PMX53 | Chymotrypsin-like elastase family member 1 -Cela1- |
| Q9CQ52 | 150.3 | 0.0006 | 23.81 | Agonist | PMX53 | Chymotrypsin-like elastase family member 3B -Cela3b- |
| Q9CR35 | 199.0 | 9.2E-09 | 21.14 | Agonist | PMX53 | Chymotrypsinogen B -Ctrb1- |
| Q3SYP2 | 45.5 | 4.0E-07 | 32.06 | Agonist | PMX53 | Chymotrypsin-C -Ctrc- |
| Q9D6Z6 | 5.9 | 6.8E-08 | Infinity | Agonist | PMX53 | Interleukin-36 beta -Il36b- |
| Q8R460 | 19.2 | 4.0E-07 | 10.08 | Agonist | PMX53 | Interleukin-36 gamma -Il36g- |
| Q9QYY1 | 45.8 | 3.7E-05 | 4.89 | Agonist | PMX53 | Interleukin-36 receptor antagonist protein -IL36RN- |
| Q9JLA2 | 46.8 | 0.0003 | 21.89 | Agonist | PMX53 | Interleukin-36 alpha -Il36a- |
| *Macrophage related markers* | | | | | | |
| Q61830 | 23.2 | 4.6E-06 | 3.71 | Agonist | PMX53 | Macrophage mannose receptor 1 -Mrc1- |
| P12388 | 73.4 | 2.3E-05 | 12.60 | Agonist | PMX53 | Plasminogen activator inhibitor 2 -Serpinb2- |
| P24452 | 68.9 | 0.001 | 1.47 | Agonist | PMX53 | Macrophage-capping protein -Capg- |
| P34884 | 39.9 | 0.02 | 1.41 | Agonist | PMX53 | Macrophage migration inhibitory factor -Mif- |
| *Complement markers* | | | | | | |
| Q8BH35 | 36.0 | 3.5E-06 | 9.54 | Agonist | PMX53 | Complement component C8 beta chain -C8b- |
| P03953 | 48.3 | 2.9E-05 | 5.01 | Agonist | PMX53 | Complement factor D -Cfd- |
| P06683 | 18.9 | 0.0003 | 25.02 | Agonist | PMX53 | Complement component C9 -C9- |
| P04186 | 64.3 | 0.0005 | 2.44 | Agonist | PMX53 | Complement factor B -Cfb- |
| P01027 | 421.1 | 0.002 | 2.58 | Agonist | PMX53 | Complement C3 -C3- |
| P01029 | 61.8 | 0.003 | 6.37 | Agonist | PMX53 | Complement C4-B -C4b- |
| Q8K182 | 23.8 | 0.005 | 9.50 | Agonist | PMX53 | Complement component C8 alpha chain -C8a- |
| P06909 | 192.8 | 0.01 | 1.28 | Agonist | PMX53 | Complement factor H -Cfh- |
| P11680 | 12.0 | 1.9E-05 | 2.96 | Agonist | PMX53 | Properdin -Cfp- |

S1 Table Phagocytic cell and complement related markers obtained through LC-MS/MS
