# Supplementary material for: Pharmacological Stimulation of Phagocytosis Enhances Amyloid Plaque Clearance; Evidence from a Transgenic Mouse Model of ATTR Neuropathy
Source: Front Mol Neurosci. 2017 May 10;10:138. doi: 10.3389/fnmol.2017.00138 (PMC5423984; doi:10.3389/fnmol.2017.00138)
Supplement: Supplementary file 2 [file Table_2.docx]

| Accession | Confidence score | Anova (p) | Max fold change | Highest mean condition | Lowest mean condition | Description |
| --- | --- | --- | --- | --- | --- | --- |
| *ER stress related markers* | | | | | | |
| P20029 | 567.6 | 8.9E-06 | 3.06 | PMX53 | Agonist | 78 kDa glucose-regulated protein -Hspa5- |
| P63038 | 617.2 | 1.1E-05 | 2.25 | PMX53 | Agonist | 60 kDa heat shock protein_ mitochondrial -Hspd1- |
| P38647 | 438.9 | 1.2E-05 | 2.65 | PMX53 | Agonist | Stress-70 protein_ mitochondrial -Hspa9- |
| P07901 | 441.1 | 0.0001 | 1.70 | PMX53 | Agonist | Heat shock protein HSP 90-alpha -Hsp90aa1- |
| Q8K0U4 | 27.0 | 0.0002 | 1.95 | PMX53 | Agonist | Heat shock 70 kDa protein 12A -Hspa12a- |
| P11499 | 618.3 | 0.0005 | 1.16 | PMX53 | Agonist | Heat shock protein HSP 90-beta -Hsp90ab1- |
| Q64433 | 98.2 | 0.001 | 1.58 | PMX53 | Agonist | 10 kDa heat shock protein_ mitochondrial -Hspe1- |
| P48722 | 91.4 | 0.002 | 2.71 | PMX53 | Agonist | Heat shock 70 kDa protein 4L -Hspa4l- |
| P63017 | 548.5 | 0.003 | 1.25 | PMX53 | Agonist | Heat shock cognate 71 kDa protein -Hspa8- |
| Q61316 | 313.1 | 0.005 | 1.30 | PMX53 | Agonist | Heat shock 70 kDa protein 4 -Hspa4- |
| *Apoptotic markers* | | | | | | |
| Q61160 | 6.6 | 0.0035 | 1.76 | PMX53 | Agonist | FAS-associated death domain protein -Fadd- |
| P70677 | 57.0 | 4.0E-05 | 3.73 | PMX53 | Agonist | Caspase-3 -Casp3- |
| O08738 | 63.7 | 0.0004 | 2.94 | PMX53 | Agonist | Caspase-6 -Casp6- |
| P97864 | 6.1 | 7.2E-05 | 3.44 | PMX53 | Agonist | Caspase-7 -Casp7- |
| O89110 | 11.1 | 0.002 | 2.32 | PMX53 | Agonist | Caspase-8 -Casp8- |

S2 Table Cellular stress and apoptosis related markers obtained through LC-MS/MS
