# Supplementary material for: Pharmacological Stimulation of Phagocytosis Enhances Amyloid Plaque Clearance; Evidence from a Transgenic Mouse Model of ATTR Neuropathy
Source: Front Mol Neurosci. 2017 May 10;10:138. doi: 10.3389/fnmol.2017.00138 (PMC5423984; doi:10.3389/fnmol.2017.00138)
Supplement: Supplementary file 3 [file Table_3.docx]

S3 Table. Proteins involved with macrophage activation

| Accession | Confidence score | Anova (p) | Max fold change | Highest mean condition | Lowest mean condition | Description |
| --- | --- | --- | --- | --- | --- | --- |
| *Macrophage Activation* | | | | | | |
| P55065 | 27,9 | 0,001 | 14,39 | AGONIST | PMX53 | Phospholipid transfer protein -Pltp- |
| Q61830 | 23,2 | 2,3E-05 | 2,37 | AGONIST | PMX53 | Macrophage mannose receptor 1 -Mrc1- |
| Q9D2Q8 | 96,1 | 9,9E-09 | 6,17 | AGONIST | PMX53 | Protein S100-A14 -S100a14- |
| P07743 | 103,7 | 5,7E-06 | 2,03 | AGONIST | PMX53 | BPI fold-containing family A member 2 -Bpifa2- |
| Q9Z0E6 | 62,6 | 0,0001 | 1,60 | AGONIST | PMX53 | Interferon-induced guanylate-binding protein 2-Gbp2- |
| P08207 | 31,7 | 0,0005 | 3,58 | AGONIST | PMX53 | Protein S100-A10 -S100a10- |
| Q01514 | 30,6 | 3,0E-05 | 3,18 | AGONIST | PMX53 | Interferon-induced guanylate-binding protein 1 -Gbp1- |
| Q60997 | 83,6 | 4,2E-05 | 4,17 | AGONIST | PMX53 | Deleted in malignant brain tumors 1 protein -Dmbt1- |
| P27005 | 37,9 | 1,9E-06 | 7,67 | AGONIST | PMX53 | Protein S100-A8 -S100a8- |
| P50543 | 35,5 | 0,0004 | 2,14 | AGONIST | PMX53 | Protein S100-A11 -S100a11- |
| Q04857 | 472,7 | 3,0E-06 | 7,12 | AGONIST | PMX53 | Collagen alpha-1(VI) chain/Col6a1 |
| Q60767 | 44,5 | 6,1E-05 | 1,81 | AGONIST | PMX53 | Lymphocyte antigen 75/Ly75 |
| Q91YH5 | 106,7 | 1,2E-05 | 1,88 | AGONIST | PMX53 | Atlastin-3/Atl3 |
| P56565 | 22,8 | 0,0006 | 2,50 | PMX53 | AGONIST | Protein S100-A1 -S100a1- |
| Q08857 | 26,0 | 0,003 | 2,11 | PMX53 | AGONIST | Platelet glycoprotein 4 -Cd36- |
