# Supplementary material for: Pharmacological Stimulation of Phagocytosis Enhances Amyloid Plaque Clearance; Evidence from a Transgenic Mouse Model of ATTR Neuropathy
Source: Front Mol Neurosci. 2017 May 10;10:138. doi: 10.3389/fnmol.2017.00138 (PMC5423984; doi:10.3389/fnmol.2017.00138)
Supplement: Supplementary file 4 [file Table_4.docx]

S4 Table. Proteins involved with complement activation

| Accession | Confidence score | Anova (p) | Max fold change | Highest mean condition | Lowest mean condition | Description |
| --- | --- | --- | --- | --- | --- | --- |
| *Complement Activation* | | | | | | |
| P01027 | 414,5 | 2,4E-05 | 2,64 | AGONIST | PMX53 | Complement C3 -C3- |
| P18531 | 5,6 | 0,005 | 116,76 | AGONIST | PMX53 | Ig heavy chain V region 3-6 -Ighv3-6- |
| P01872 | 153,4 | 2,4E-08 | 3,06 | AGONIST | PMX53 | Ig mu chain C region -Ighm- |
| P41317 | 12,8 | 0,0003 | 4,63 | AGONIST | PMX53 | Mannose-binding protein C -Mbl2- |
| P01867 | 99,1 | 0,0002 | 4,17 | AGONIST | PMX53 | Ig gamma-2B chain C region -Igh-3- |
| Q8R422 | 29,0 | 0,002 | 2,09 | AGONIST | PMX53 | CD109 antigen -Cd109- |
| P01837 | 52,3 | 1,2E-05 | 2,30 | AGONIST | PMX53 | Ig kappa chain C region -unassigned- |
| Q61838 | 658,6 | 3,2E-06 | 6,63 | AGONIST | PMX53 | Pregnancy zone protein -Pzp- |
| P04186 | 64,4 | 0,0005 | 2,44 | AGONIST | PMX53 | Complement factor B -Cfb- |
| P01029 | 62,0 | 0,003 | 6,37 | AGONIST | PMX53 | Complement C4-B -C4b- |
| Q8K182 | 18,2 | 0,005 | 9,50 | AGONIST | PMX53 | Complement component C8 alpha chain -C8a- |
| Q3UU35 | 90,6 | 0,0001 | 2,18 | AGONIST | PMX53 | Ovostatin homolog -Ovos- |
| P08607 | 13,3 | 0,0001 | 8,28 | PMX53 | AGONIST | C4b-binding protein -C4bpa- |
