# Supplementary material for: Pharmacological Stimulation of Phagocytosis Enhances Amyloid Plaque Clearance; Evidence from a Transgenic Mouse Model of ATTR Neuropathy
Source: Front Mol Neurosci. 2017 May 10;10:138. doi: 10.3389/fnmol.2017.00138 (PMC5423984; doi:10.3389/fnmol.2017.00138)
Supplement: Supplementary file 5 [file Table_5.docx]

S5 Table. Proteins involved with apoptosis

| Accession | Confidence score | Anova (p) | Max fold change | Highest mean condition | Lowest mean condition | Description |
| --- | --- | --- | --- | --- | --- | --- |
| *Apoptosis* | | | | | | |
| P16627 | 221,1 | 0,0006 | 1,84 | AGONIST | PMX53 | Heat shock 70 kDa protein 1-like -Hspa1l- |
| P17879 | 438,0 | 0,0002 | 2,60 | AGONIST | PMX53 | Heat shock 70 kDa protein 1B -Hspa1b- |
| Q61831 | 28,3 | 0,0004 | 1,70 | AGONIST | PMX53 | Mitogen-activated protein kinase 10 -Mapk10- |
| Q61696 | 437,9 | 0,0002 | 2,60 | AGONIST | PMX53 | Heat shock 70 kDa protein 1A -Hspa1a- |
| Q9JLV1 | 76,0 | 0,006 | 2,09 | AGONIST | PMX53 | BAG family molecular chaperone regulator 3 -Bag3- |
| P25799 | 18,7 | 1,2E-05 | 3,13 | AGONIST | PMX53 | Nuclear factor NF-kappa-B p105 subunit -Nfkb1- |
| P24452 | 68,9 | 0,001 | 1,47 | AGONIST | PMX53 | Macrophage-capping protein -Capg- |
| P48678 | 638,4 | 4,9E-06 | 2,02 | AGONIST | PMX53 | Prelamin-A/C -Lmna- |
| Q9WTX2 | 5,8 | 0,04 | 1,64 | PMX53 | AGONIST | Interferon-inducible double-stranded RNA-dependent protein kinase activator A -Prkra- |
| P97864 | 6,1 | 7,2E-05 | 3,44 | PMX53 | AGONIST | Caspase-7 -Casp7- |
| P70677 | 38,9 | 0,002 | 1,63 | PMX53 | AGONIST | Caspase-3 -Casp3- |
| P28867 | 22,5 | 9,2E-05 | 1,78 | PMX53 | AGONIST | Protein kinase C delta type -Prkcd- |
| Q3U0V2 | 5,8 | 0,006 | 3,15 | PMX53 | AGONIST | Tumor necrosis factor receptor type 1-associated DEATH domain protein -Tradd- |
| P47809 | 12,3 | 0,03 | 1,47 | PMX53 | AGONIST | Dual specificity mitogen-activated protein kinase kinase  -Map2k4- |
| Q9WTU6 | 28,0 | 2,0E-05 | 29,88 | PMX53 | AGONIST | Mitogen-activated protein kinase 9 -Mapk9- |
| Q61160 | 6,6 | 0,003 | 1,76 | PMX53 | AGONIST | FAS-associated death domain protein -Fadd- |
| Q60739 | 12,4 | 5,8E-05 | 2,18 | PMX53 | AGONIST | BAG family molecular chaperone regulator 1 -Bag1- |
| O89110 | 11,1 | 0,001 | 2,32 | PMX53 | AGONIST | Caspase-8 -Casp8- |
| P11103 | 23,9 | 0,003 | 2,03 | PMX53 | AGONIST | Poly [ADP-ribose] polymerase 1 -Parp1- |
| O08738 | 80,7 | 0,001 | 3,24 | PMX53 | AGONIST | Caspase-6 -Casp6 - |
| Q9D0M3 | 100,5 | 4,3E-06 | 4,61 | PMX53 | AGONIST | Cytochrome c1_ heme protein_ mitochondrial -Cyc1- |
