# Supplementary material for: Pharmacological Stimulation of Phagocytosis Enhances Amyloid Plaque Clearance; Evidence from a Transgenic Mouse Model of ATTR Neuropathy
Source: Front Mol Neurosci. 2017 May 10;10:138. doi: 10.3389/fnmol.2017.00138 (PMC5423984; doi:10.3389/fnmol.2017.00138)
Supplement: Supplementary file 6 [file Table_6.docx]

S6 Table. Proteins involved with inflammation mediated by cytokines and chemokines (1/2)

| Accession | Confidence score | Anova (p) | Max fold change | Highest mean condition | Lowest mean condition | Description |
| --- | --- | --- | --- | --- | --- | --- |
| *Inflammation mediated by cytokines and chemokines (1/2)* | | | | | | |
| P10833 | 86,0 | 0,002 | 1,54 | AGONIST | PMX53 | Ras-related protein R-Ras -Rras- |
| P39655 | 11,8 | 0,0002 | 2,65 | AGONIST | PMX53 | Arachidonate 12-lipoxygenase, 12S-type -Alox12- |
| Q8C6K9 | 129,8 | 0,0008 | 1,51 | AGONIST | PMX53 | Collagen alpha-6(VI) chain -Col6a6- |
| Q923T9 | 33,0 | 5,3E-05 | 2,99 | AGONIST | PMX53 | Calcium/calmodulin-dependent protein kinase type II subunit gamma -Camk2g- |
| Q61879 | 477,5 | 7,5E-05 | 2,60 | AGONIST | PMX53 | Myosin-10 -Myh10- |
| P70380 | 6,2 | 2,6E-05 | 2,61 | AGONIST | PMX53 | Interleukin-18 -Il18- |
| P0C871 | 18,6 | 8,8E-06 | 24,25 | AGONIST | PMX53 | Cytosolic phospholipase A2 beta -Pla2g4b- |
| Q8R3B1 | 106,5 | 7,9E-05 | 3,51 | AGONIST | PMX53 | 1-phosphatidylinositol 4,5-bisphosphate phosphodiesterase delta-1 -Plcd1- |
| Q02788 | 321,0 | 3,0E-06 | 7,38 | AGONIST | PMX53 | Collagen alpha-2(VI) chain -Col6a2- |
| Q9JLA2 | 46,8 | 0,0003 | 21,89 | AGONIST | PMX53 | Interleukin-36 alpha -Il36a- |
| Q04857 | 472,7 | 3,0E-06 | 7,12 | AGONIST | PMX53 | Collagen alpha-1(VI) chain -Col6a1- |
| Q99MK8 | 0 | 0,002 | 2,16 | AGONIST | PMX53 | Beta-adrenergic receptor kinase 1 -Adrbk1- |
| O08638 | 2462,8 | 1,3E-06 | 3,26 | AGONIST | PMX53 | Myosin-11 -Myh11- |
| Q6URW6 | 379,6 | 0,02 | 1,46 | AGONIST | PMX53 | Myosin-14 -Myh14- |
| P42225 | 11,5 | 0,0005 | 3,59 | AGONIST | PMX53 | Signal transducer and activator of transcription 1 -Stat1- |
| P25799 | 18,7 | 1,2E-05 | 3,13 | AGONIST | PMX53 | Nuclear factor NF-kappa-B p105 subunit -Nfkb1- |
| O08648 | 10,9 | 0,0002 | 2,36 | AGONIST | PMX53 | Mitogen-activated protein kinase kinase kinase 4 -Map3k4- |
| Q8VDD5 | 1203,8 | 0,0001 | 1,92 | AGONIST | PMX53 | Myosin-9 -Myh9- |
| Q80X19 | 192,6 | 0,0005 | 1,45 | AGONIST | PMX53 | Collagen alpha-1(XIV) chain -Col14a1- |
| P09055 | 265,8 | 1,3E-05 | 2,51 | AGONIST | PMX53 | Integrin beta-1 -Itgb1- |
| Q61831 | 28,3 | 0,0004 | 1,70 | AGONIST | PMX53 | Mitogen-activated protein kinase 10 -Mapk10- |
| P47811 | 55,9 | 0,01 | 1,71 | AGONIST | PMX53 | Mitogen-activated protein kinase 14 -Mapk14- |
| Q9D6Z6 | 6,0 | 6,8E-08 | Infinity | AGONIST | PMX53 | Interleukin-36 beta -Il36b- |

S6 Table. Proteins involved with inflammation mediated by cytokines and chemokines (2/2)

| Accession | Confidence score | Anova (p) | Max fold change | Highest mean condition | Lowest mean condition | Description |
| --- | --- | --- | --- | --- | --- | --- |
| *Inflammation mediated by cytokines and chemokines (2/2)* | | | | | | |
| Q9R0Q6 | 65,0 | 7,2E-06 | 2,99 | PMX53 | AGONIST | Actin-related protein 2/3 complex subunit 1A -Arpc1a- |
| P18872 | 65,7 | 0,0002 | 2,61 | PMX53 | AGONIST | Guanine nucleotide-binding protein G(o) subunit alpha -Gnao1- |
| O88643 | 45,9 | 0,0002 | 3,77 | PMX53 | AGONIST | Serine/threonine-protein kinase PAK 1 -Pak1- |
| Q9CVB6 | 163,5 | 0,0001 | 1,45 | PMX53 | AGONIST | Actin-related protein 2/3 complex subunit 2 -Arpc2- |
| P59999 | 54,5 | 0,0002 | 1,59 | PMX53 | AGONIST | Actin-related protein 2/3 complex subunit 4 -Arpc4- |
| Q8K3H5 | 24,7 | 5,4E-07 | 11,86 | PMX53 | AGONIST | Myosin-IIIa -Myo3a- |
| P70227 | 52,3 | 2,7E-06 | 2,32 | PMX53 | AGONIST | Inositol 1,4,5-trisphosphate receptor type 3 -Itpr3- |
| Q8CIN4 | 83,3 | 0,0004 | 1,51 | PMX53 | AGONIST | Serine/threonine-protein kinase PAK 2 -Pak2- |
| P21278 | 65,4 | 5,6E-05 | 1,88 | PMX53 | AGONIST | Guanine nucleotide-binding protein subunit alpha-11 -Gna11- |
| O70589 | 24,1 | 0,0004 | 2,26 | PMX53 | AGONIST | Peripheral plasma membrane protein CASK -Cask- |
| Q62159 | 66,5 | 0,0004 | 1,45 | PMX53 | AGONIST | Rho-related GTP-binding protein RhoC -Rhoc- |
| Q9WTU6 | 28,0 | 2,0E-05 | 29,88 | PMX53 | AGONIST | Mitogen-activated protein kinase 9 -Mapk9- |
| Q9Z1B7 | 22,7 | 2,9E-05 | 2,35 | PMX53 | AGONIST | Mitogen-activated protein kinase 13 -Mapk13- |
| P18653 | 35,5 | 0,003 | 3,31 | PMX53 | AGONIST | Ribosomal protein S6 kinase alpha-1 -Rps6ka1- |
