# Supplementary material for: Pharmacological Stimulation of Phagocytosis Enhances Amyloid Plaque Clearance; Evidence from a Transgenic Mouse Model of ATTR Neuropathy
Source: Front Mol Neurosci. 2017 May 10;10:138. doi: 10.3389/fnmol.2017.00138 (PMC5423984; doi:10.3389/fnmol.2017.00138)
Supplement: Supplementary file 7 [file Table_7.docx]

S7 Table. Proteins involved with peptidase Activity (1/4)

| Accession | Confidence score | Anova (p) | Max fold change | Highest mean condition | Lowest mean condition | Description |
| --- | --- | --- | --- | --- | --- | --- |
| *Peptidase Activity (1/4)* | | | | | | |
| P05208 | 154,6 | 2,6E-05 | 34,80 | AGONIST | PMX53 | Chymotrypsin-like elastase family member 2A -Cela2a- |
| Q640N1 | 17,7 | 0,001 | 2,85 | AGONIST | PMX53 | Adipocyte enhancer-binding protein 1 -Aebp1- |
| O08663 | 47,9 | 0,003 | 1,81 | AGONIST | PMX53 | Methionine aminopeptidase 2 -Metap2- |
| Q6P8K8 | 34,3 | 0,005 | 3,38 | AGONIST | PMX53 | Carboxypeptidase A4 -Cpa4- |
| P01027 | 414,5 | 2,4E-05 | 2,64 | AGONIST | PMX53 | Complement C3 -C3- |
| Q91X79 | 146,3 | 6,7E-05 | 4,86 | AGONIST | PMX53 | Chymotrypsin-like elastase family member 1 -Cela1- |
| Q9JHR7 | 270,9 | 4,6E-06 | 2,13 | AGONIST | PMX53 | Insulin-degrading enzyme -Ide- |
| Q9ESB3 | 44,3 | 6,6E-05 | 1,93 | AGONIST | PMX53 | Histidine-rich glycoprotein -Hrg- |
| P19221 | 102,0 | 0,0005 | 1,60 | AGONIST | PMX53 | Prothrombin -F2- |
| Q99JR5 | 92,6 | 0,03 | 2,37 | AGONIST | PMX53 | Tubulointerstitial nephritis antigen-like -Tinagl1- |
| Q9D7P9 | 446,0 | 3,0E-07 | 14,78 | AGONIST | PMX53 | Serpin B12 -Serpinb12- |
| A6X935 | 75,1 | 0,0002 | 1,87 | AGONIST | PMX53 | Inter alpha-trypsin inhibitor, heavy chain 4 -Itih4- |
| O08677 | 136,3 | 2,7E-06 | 3,32 | AGONIST | PMX53 | Kininogen-1 -Kng1- |
| Q61704 | 54,5 | 1,8E-06 | 3,75 | AGONIST | PMX53 | Inter-alpha-trypsin inhibitor heavy chain H3 -Itih3- |
| Q00897 | 215,9 | 4,1E-05 | 3,73 | AGONIST | PMX53 | Alpha-1-antitrypsin 1-4 -Serpina1d- |
| O08692 | 7,3 | 0,004 | 5,63 | AGONIST | PMX53 | Neutrophilic granule protein -Ngp- |
| Q7TMF5 | 31,6 | 2,2E-07 | 5,24 | AGONIST | PMX53 | Serpin A12 -Serpina12- |
| Q9QWK4 | 25,3 | 4,4E-05 | 3,62 | AGONIST | PMX53 | CD5 antigen-like -Cd5l- |
| Q60997 | 83,6 | 4,2E-05 | 4,17 | AGONIST | PMX53 | Deleted in malignant brain tumors 1 protein -Dmbt1- |
| Q09PK2 | 214,9 | 1,5E-06 | 41,53 | AGONIST | PMX53 | Retroviral-like aspartic protease 1 -Asprv1- |
| Q6NXK7 | 5,2 | 0,007 | 11,58 | AGONIST | PMX53 | Inactive dipeptidyl peptidase 10 -Dpp10- |
| P56567 | 49,9 | 0,0003 | 10,87 | AGONIST | PMX53 | Cystatin-A -Csta- |
| P06797 | 89,3 | 0,001 | 7,33 | AGONIST | PMX53 | Cathepsin L1 -Ctsl- |
| Q504N0 | 58,2 | 0,0004 | 8,56 | AGONIST | PMX53 | Carboxypeptidase A2 -Cpa2- |

S7 Table. Proteins involved with peptidase Activity (2/4)

| Accession | Confidence score | Anova (p) | Max fold change | Highest mean condition | Lowest mean condition | Description |
| --- | --- | --- | --- | --- | --- | --- |
| *Peptidase Activity (2/4)* | | | | | | |
| P12388 | 73,4 | 2,3E-05 | 12,60 | AGONIST | PMX53 | Plasminogen activator inhibitor 2, macrophage -Serpinb2- |
| P35175 | 24,2 | 4,6E-05 | 6,26 | AGONIST | PMX53 | Stefin-1 -Stfa1- |
| Q8R422 | 29,0 | 0,002 | 2,09 | AGONIST | PMX53 | CD109 antigen -Cd109- |
| Q9EPB4 | 122,0 | 0,0001 | 3,85 | AGONIST | PMX53 | Apoptosis-associated speck-like protein containing a CARD  -Pycard- |
| P35173 | 6,6 | 0,001 | 1,89 | AGONIST | PMX53 | Stefin-3 -Stfa3- |
| Q8C1A5 | 164,0 | 0,0002 | 1,71 | AGONIST | PMX53 | Thimet oligopeptidase -Thop1- |
| P07146 | 118,6 | 1,2E-07 | 15,03 | AGONIST | PMX53 | Anionic trypsin-2 -Prss2- |
| Q61838 | 658,6 | 3,2E-06 | 6,63 | AGONIST | PMX53 | Alpha-2-macroglobulin -A2m- |
| P21844 | 79,6 | 5,7E-06 | 10,00 | AGONIST | PMX53 | Chymase -Cma1- |
| Q8BFY6 | 39,4 | 0,002 | 1,71 | AGONIST | PMX53 | Peflin -Pef1- |
| Q921Z5 | 6,6 | 0,005 | 3,34 | AGONIST | PMX53 | Tumor necrosis factor alpha-induced protein 8 -Tnfaip8- |
| Q7TMR0 | 17,4 | 3,0E-05 | 6,13 | AGONIST | PMX53 | Lysosomal Pro-X carboxypeptidase -Prcp- |
| P51125 | 145,8 | 8,9E-05 | 2,26 | AGONIST | PMX53 | Calpastatin -Cast- |
| P97298 | 11,2 | 0,001 | 14,83 | AGONIST | PMX53 | Pigment epithelium-derived factor -Serpinf1- |
| Q06770 | 36,0 | 4,1E-05 | 2,17 | AGONIST | PMX53 | Corticosteroid-binding globulin -Serpina6- |
| Q9CQ52 | 150,1 | 0,0006 | 23,81 | AGONIST | PMX53 | Chymotrypsin-like elastase family member 3B -Cela3b- |
| P29699 | 148,8 | 7,7E-05 | 2,65 | AGONIST | PMX53 | Alpha-2-HS-glycoprotein -Ahsg- |
| P20918 | 143,5 | 1,3E-05 | 10,07 | AGONIST | PMX53 | Plasminogen -Plg- |
| Q07235 | 8,4 | 0,002 | 78,78 | AGONIST | PMX53 | Glia-derived nexin -Serpine2- |
| O89094 | 98,0 | 1,1E-08 | 13,68 | AGONIST | PMX53 | Caspase-14 -Casp14- |
| Q9CR35 | 199,0 | 9,2E-09 | 21,14 | AGONIST | PMX53 | Chymotrypsinogen B -Ctrb1- |
| Q7TPZ8 | 164,5 | 0,0001 | 13,97 | AGONIST | PMX53 | Carboxypeptidase A1 -Cpa1- |
| Q9Z0L8 | 83,8 | 1,7E-05 | 4,13 | AGONIST | PMX53 | Gamma-glutamyl hydrolase -Ggh- |
| P01029 | 62,0 | 0,003 | 6,37 | AGONIST | PMX53 | Complement C4-B -C4b- |

S7 Table. Proteins involved with peptidase Activity (3/4)

| Accession | Confidence score | Anova (p) | Max fold change | Highest mean condition | Lowest mean condition | Description |
| --- | --- | --- | --- | --- | --- | --- |
| *Peptidase Activity (3/4)* | | | | | | |
| Q61703 | 41,6 | 6,2E-06 | 1,96 | AGONIST | PMX53 | Inter-alpha-trypsin inhibitor heavy chain H2 -Itih2- |
| Q61247 | 32,0 | 0,0002 | 2,60 | AGONIST | PMX53 | Alpha-2-antiplasmin -Serpinf2- |
| P15089 | 54,6 | 2,4E-06 | 34,00 | AGONIST | PMX53 | Mast cell carboxypeptidase A -Cpa3- |
| Q8K1K6 | 30,2 | 0,001 | 1,65 | AGONIST | PMX53 | Serpin B10 -Serpinb10- |
| Q9QXC1 | 128,7 | 7,6E-06 | 5,81 | AGONIST | PMX53 | Fetuin-B -Fetub- |
| Q3UU35 | 90,6 | 0,0001 | 2,18 | AGONIST | PMX53 | Ovostatin homolog -Ovos- |
| P97290 | 25,9 | 0,001 | 1,67 | AGONIST | PMX53 | Plasma protease C1 inhibitor -Serping1- |
| P70124 | 221,2 | 2,30E-05 | 1,91 | AGONIST | PMX53 | Serpin B5 -Serpinb5- |
| Q9CZ13 | 311,9 | 3,60E-05 | 3,79 | PMX53 | AGONIST | Cytochrome b-c1 complex subunit 1, mitochondrial -Uqcrc1- |
| Q3TXS7 | 161,7 | 0,0002 | 1,62 | PMX53 | AGONIST | 26S proteasome non-ATPase regulatory subunit 1 -Psmd1- |
| O89023 | 45,6 | 0,005 | 2,50 | PMX53 | AGONIST | Tripeptidyl-peptidase 1 -Tpp1- |
| P97864 | 6,1 | 7,2E-05 | 3,44 | PMX53 | AGONIST | Caspase-7 -Casp7- |
| Q8VCT3 | 181,2 | 1,8E-05 | 2,30 | PMX53 | AGONIST | Aminopeptidase B -Rnpep- |
| O08738 | 80,7 | 0,001 | 3,24 | PMX53 | AGONIST | Caspase-6 -Casp6- |
| Q99JW2 | 71,0 | 2,0E-05 | 2,46 | PMX53 | AGONIST | Aminoacylase-1 -Acy1- |
| P16675 | 68,8 | 0,0007 | 1,86 | PMX53 | AGONIST | Lysosomal protective protein -Ctsa- |
| Q8C129 | 37,9 | 0,0002 | 3,34 | PMX53 | AGONIST | Leucyl-cystinyl aminopeptidase -Lnpep- |
| Q8C0M9 | 12,2 | 0,003 | 1,62 | PMX53 | AGONIST | Isoaspartyl peptidase/L-asparaginase -Asrgl1- |
| Q8JZQ2 | 24,2 | 9,3E-06 | 3,74 | PMX53 | AGONIST | AFG3-like protein 2 -Afg3l2- |
| Q60854 | 158,8 | 0,004 | 1,70 | PMX53 | AGONIST | Serpin B6 -Serpinb6- |
| P15119 | 40,8 | 0,0008 | 2,08 | PMX53 | AGONIST | Mast cell protease 2 -Mcpt2- |
| Q6P069 | 76,6 | 2,6E-05 | 2,53 | PMX53 | AGONIST | Sorcin -Sri- |
| P31428 | 17,1 | 0,0002 | 2,01 | PMX53 | AGONIST | Dipeptidase 1 -Dpep1- |
| Q9R013 | 17,5 | 1,7E-05 | 2,55 | PMX53 | AGONIST | Cathepsin F-Ctsf- |

S7 Table. Proteins involved with peptidase Activity (4/4)

| Accession | Confidence score | Anova (p) | Max fold change | Highest mean condition | Lowest mean condition | Description |
| --- | --- | --- | --- | --- | --- | --- |
| *Peptidase Activity (4/4)* | | | | | | |
| P18242 | 288,8 | 4,4E-05 | 2,26 | PMX53 | AGONIST | Cathepsin D -Ctsd- |
| Q8R146 | 74,6 | 0,004 | 1,79 | PMX53 | AGONIST | Acylamino-acid-releasing enzyme -Apeh- |
| P70677 | 38,9 | 0,002 | 1,63 | PMX53 | AGONIST | Caspase-3 -Casp3- |
| O35350 | 87,9 | 0,0001 | 1,50 | PMX53 | AGONIST | Calpain-1 catalytic subunit -Capn1- |
| P10605 | 155,5 | 5,0E-05 | 1,83 | PMX53 | AGONIST | Cathepsin B -Ctsb- |
| Q91VA3 | 10,8 | 0,002 | 1,84 | PMX53 | AGONIST | Calpain-8 -Capn8- |
| O88544 | 93,7 | 0,0008 | 1,59 | PMX53 | AGONIST | COP9 signalosome complex subunit 4 -Cops4- |
| P97449 | 84,1 | 0,0006 | 1,87 | PMX53 | AGONIST | Aminopeptidase N -Anpep- |
| Q9ET22 | 42,8 | 2,8E-07 | 6,36 | PMX53 | AGONIST | Dipeptidyl peptidase 2 -Dpp7- |
| Q9D7R7 | 36,0 | 4,5E-05 | 12,71 | PMX53 | AGONIST | Gastricsin -Pgc- |
| Q91YP2 | 22,9 | 0,005 | 1,70 | PMX53 | AGONIST | Neurolysin, mitochondrial -Nln- |
| Q8CIN4 | 83,3 | 0,0004 | 1,51 | PMX53 | AGONIST | Serine/threonine-protein kinase PAK 2 -Pak2- |
| Q9CXT8 | 32,7 | 0,001 | 2,24 | PMX53 | AGONIST | Mitochondrial-processing peptidase subunit beta -Pmpcb- |
| Q9QXV0 | 19,7 | 0,0003 | 1,72 | PMX53 | AGONIST | ProSAAS -Pcsk1n- |
| Q9DC61 | 24,2 | 0,005 | 2,05 | PMX53 | AGONIST | Mitochondrial-processing peptidase subunit alpha -Pmpca- |
| Q99K23 | 17,4 | 5,7E-05 | 4,01 | PMX53 | AGONIST | Ufm1-specific protease 2 -Ufsp2- |
| Q00493 | 78,2 | 0,0005 | 1,68 | PMX53 | AGONIST | Carboxypeptidase E -Cpe- |
| O89110 | 11,1 | 0,001 | 2,32 | PMX53 | AGONIST | Caspase-8 -Casp8- |
| P24527 | 177,3 | 3,4E-05 | 2,41 | PMX53 | AGONIST | Leukotriene A-4 hydrolase -Lta4h- |
| P23578 | 13,5 | 4,0E-06 | 7,90 | PMX53 | AGONIST | Acrosin -Acr- |
| P70269 | 57,6 | 0,02 | 3,38 | PMX53 | AGONIST | Cathepsin E -Ctse- |
